# Evaluation of spatial variation in chronic wasting disease risk with Bayesian Poisson log-Gaussian model

**DOI:** 10.3389/fvets.2025.1568468

**Published:** 2025-11-07

**Authors:** Ram K. Raghavan, Frank Badu Osei, Alfred Stein, Shane Hesting, Levi Jaster, Bijaya Hatuwal, Joseph E. Mosley, Akila Raghavan

**Affiliations:** 1 Department of Pathobiology and Integrative Biomedical Sciences, College of Veterinary Medicine, University of Missouri, Columbia, MO, United States; 2 Department of Public Health, College of Health Sciences, University of Missouri, Columbia, MO, United States; 3 MU Institute of Data Science and Informatics, University of Missouri, Columbia, MO, United States; 4 Faculty of Geo-Information Science and Earth Observation (ITC), University of Twente, Enschede, Netherlands; 5 Kansas Department of Wildlife & Parks, Emporia, KS, United States; 6 Department of Electrical Engineering and Computer Science, University of Missouri, Columbia, MO, United States

**Keywords:** chronic wasting disease, white-tailed deer, mule deer, Poisson log-Gaussian model, rare disease

## Abstract

Chronic wasting disease (CWD) is a fatal neurodegenerative disease among cervids that has steadily spread across the United States and Canada. The year-to-year increase in the geographic spread of this disease among white-tailed deer and mule deer have raised concerns about conserving these species and sustainable big-game hunting. Knowledge of the spatial variation in CWD risk in Kansas, a state that attracts big game hunters nationwide is not fully understood. We explored the spatial variation in CWD risk and the potential effects of habitat-level covariates using surveillance data collected in Kansas from 2005 to 2023, with a Poisson log-Gaussian model in a Bayesian framework. Two models were considered; Model 1 included only spatial random effects and Model 2 included spatial random effects plus non-linear effects of habitat-level covariate. Following satisfactory convergence of model parameters, choropleth maps of posterior mean estimates for the risk of CWD presence, and measures of spatial heterogeneities were plotted. The impacts of the habitat-level covariates were deemed important predictors of CWD as Model 2 outperformed Model 1. The risk of CWD in the northwestern and southcentral portions of the state is likely driven by similar underlying spatial processes; however, no global smoothing effect was observed. The northwestern region is at higher risk for CWD presence but a gradual increase in risk toward the south and eastern sides of the state is apparent. We conclude that the data-driven Poisson log-Gaussian model is useful in assessing CWD and potentially other wildlife diseases from surveillance sources, and the different spatial patterns and habitat-level covariate association have relevance to CWD management in Kansas.

## 1 Introduction

Chronic wasting disease (CWD) is a neurodegenerative prion disease of cervids that has steadily increased in its spatial distribution in N. America since it was first described among wild deer in 1981 (1, 2). Its spread and establishment among white-tailed deer (*Odocoileus virginianus*) and other common cervid species threaten big-game hunting, which is often the economic backbone for many rural communities in N. America (3, 4). It has been suggested that if uncontrolled, CWD could irrevocably negatively affect cervid population stability in its enzootic region, which is currently spread across 36 states in the US and 6 provinces in Canada (5–7). Furthermore, although currently not confirmed by clinical evidence, there is a concern that CWD prions could at some point in the future adversely affect those who consume contaminated venison, as was the case with consumption of contaminated beef and bovine spongiform encephalopathy (BSE, or mad cow disease), a different prion disease (8).

To monitor and manage CWD, state wildlife agencies conduct spatiotemporal surveillance for the presence of CWD prions among different cervid species, primarily the white-tailed deer and mule deer (*Odocoileus hemionus*) but also Elk (*Cervus canadensis*), and Moose (*Alces alces*), where they are present in higher numbers. In Kansas, the study region for the present work, the Kansas Department of Wildlife and Parks (KDWP) has conducted such surveillance annually since 1996, most years during the deer hunting season (mid-November through mid-January). Surveillance in most wildlife management jurisdictions, including those in Kansas, is conducted irregularly with different focus areas each year and in a non-probabilistic manner. Diagnostic samples used in surveillance are acquired from taxidermists, meat processors, vehicle-killed deer, and hunter-harvested deer, sick/suspect deer, and private vendors (collectors) are often compensated for their efforts.

One of the goals of such surveillance is to detect the disease spatial clusters [e.g., (9)] or spatial variations in CWD risk and to identify their potential environmental drivers so that the disease can be managed efficiently. The Habitat Risk software (10) for instance allows users to derive localized predictions of CWD risk using disease testing data, the environment (land cover/land use, soil properties) and host-level factors (age, sex). The majority of the samples diagnosed through such surveillance efforts particularly outside epizootic areas, however, test negative for CWD, with only rare occurrences of positive diagnosis. These characteristics, *viz*., spatiotemporally irregular, and non-probabilistic sampling, outcome class imbalance (i.e., over-representation of one of the two diagnostic outcomes, positive or negative), low background prevalence (i.e., rare events) in some surveillance areas vs. others, among other artifacts such as spatiotemporal heterogeneity in prevalence, warrant the development of data-driven spatial models that can accommodate for such data artifacts. A Poisson log-Gaussian model is a hierarchical model used to analyze count data that exhibit overdispersion and spatial or spatiotemporal correlation. It combines a Poisson likelihood for observed counts with a latent Gaussian random field (GRF) on the log-scale of the intensity. It captures the extra-Poisson variation (overdispersion) through the latent field, and accounts for spatial autocorrelation in residual risk that are not explained by the random effects or covariates.

This objective of this study was to examine the Kansas statewide spatial variation in CWD risk and the potential contribution of habitat (land cover/land use) variables using a Poisson log-Gaussian model in a Bayesian framework. Specifically, our goals were to analyze the spatial distribution of CWD counts over regularly sized 20 km^2^ grids in Kansas with a focus on quantifying the spatial variation in risk and the effects of habitat-level covariates on the rare counts. The methods we describe here could have broad applicability for wildlife disease modeling as many such diseases share similar data characteristics.

## 2 Materials and methods

### 2.1 Data

#### 2.1.1 Disease data

Surveillance data representing the diagnostic test results of CWD presence by means of immunohistochemistry analysis (11) of tissue samples from harvested deer, corresponding species, demographic characteristics (sex, age-group), and harvest locations (approximated within 5 kms) were available from KDWP for years 2005–2018. Identical data collected by means of a surveillance project led by the authors in the present study (2019–2023) were added to this dataset and curated in sequential steps to render them ready for statistical modeling. Curation steps broadly included the exclusion of incomplete or redundant records. Subsequently, the data were geocoded with the longitude/latitude data in ArcMap 10.8.2 (ESRI, Redlands, CA). A 20 km^2^ grid was created covering the entire state of Kansas. In the present study, we grouped white-tailed deer, mule deer and the different demographic groups as a single dependent variable with potentially two outcomes for disease status; not-detected (coded 0) and detected (coded 1). Their counts were aggregated within the individual 20 km^2^ grid cells across the study region.

There were 28,754 CWD diagnostic test records in the surveillance dataset available to us, of which 316 records had test results marked as “Unknown,” and 93 records were marked “Unsuitable samples.” There were 6,494 that lacked geographic coordinates (either or both latitude and longitude), and 236 records were with coordinates outside Kansas. These records were sequentially removed, which resulted in 21,615 total unique spatially referenced records for spatial analysis. Of these, the presence of CWD was not detected in 20,905 samples (96.71%) and detected in 711 (3.28%). This dataset represents data collected during the annual deer hunting season from 2005 till 2023.

#### 2.1.2 Environmental data

Land cover and land use characteristics are good substitute variables for wildlife habitats and have been previously shown to be associated with CWD epizootiology [e.g., (12, 13)]. In an automated routine in R-Studio, we calculated the total area and proportion of different land cover classes within each grid cell from the 2016 National Land Cover Dataset (NLCD) (14). The NLCD is a source of complete, consistent information of the US land cover, released by the US Geological Survey and a consortium of US federal agencies, once approximately every 5 years. We used the 2016 dataset in the present study as it corresponded approximately with the mid-point of the surveillance period. For the study region, 13 land cover variables present in the NLCD were summarized for each grid cell (Table 1). Developed, low intensity, developed, medium intensity, and developed, high Intensity were not associated with the disease status in a univariate screening (*p* ≥ 0.2) and were removed from analysis. Additionally, the observations for the variable deciduous forest were empty in > 80% of the 20 km^2^ grid cell, and therefore not included in the analysis.

**Table 1:** Land cover classes found in 2016 National Land Cover Dataset corresponding to the study region (Kansas).

Dataset (source)	Classes
National Land Cover Dataset (MRLC)	Water; Developed, open space; Developed, low intensity; Developed, medium intensity; Developed, high intensity; Bare rock/Sand/Clay Deciduous Forest; Shrub/Scrub; Grassland/Herbaceous; Pasture/Hay; Cultivated crops; Woody wetlands; Emergent herbaceous wetlands.

### 2.2 Statistical modeling

In this study, we excluded explicit temporal random effects from the model due to irregular and inconsistent sampling of spatial units across years. Under such conditions, temporal structures are often poorly identified and can result in posterior inference that is driven more by prior assumptions than by the data (15, 16). This is especially relevant in Bayesian hierarchical models, where latent temporal terms (e.g., autoregressive or random walk priors) require sufficient and consistent coverage across both space and time to yield reliable estimates. Further, the environmental covariates included in our model are relatively stable over time, and the primary objective of the study was to characterize the long-term spatial patterns rather than short-term temporal dynamics. In such cases, incorporating temporal random effects may add complexity without improving model performance or interpretability (17). From a computational perspective, omitting the temporal component also reduces the risk of convergence issues, which are more common in complex hierarchical models with zero-inflation or sparse observations such as in our dataset (18). Focusing on spatial random effects and covariate associations, therefore, provides a more robust and interpretable framework for identifying persistent spatial heterogeneity under conditions of temporally sparse and uneven sampling. Additionally, a number of progressively complex exploratory models with host-level and surveillance source factors were constructed to quantify their potential effect on the spatial distribution of CWD in the study area. However, their inclusion did not significantly improve model performance (Supplementary File 1). Therefore, we proceeded to fit Poisson log-Gaussian spatial model as described below.

#### 2.2.1 The Poisson log-Gaussian spatial model

We fitted a Poisson log-Gaussian spatial model to (1) explore the spatial variation of CWD risk and (2) evaluate the patterns of environmental exposures on the spatial variation. We considered the CWD detected counts $y_{i}$, i=1,…,N as rare Poisson outcomes, i.e., $y_{i}\sim \text{Pois}(\mu_{i})$, with a spatially varying mean $\mu_{i}$. The intensity $\lambda_{i}$, which is controlled by spatial heterogeneity and environmental exposures is used to infer the risk of infection and is deduced as $\mu_{i}=o_{i}\lambda_{i}$


$$\log\lambda_{i}=\eta_{i}$$

$$\eta_{i}\sim N({\bar{\eta }}_{i},\sigma_{\eta }^{2})$$

$${\bar{\eta }}_{i}=\beta_{0}+\sum_{j}^{J}\beta_{j}x_{\mathrm{ij}}+\sum_{j=1}^{J}\sum_{k}^{K}\gamma_{\mathrm{jk}}{\hat{z}}_{\mathrm{ijk}}+U_{i}$$

Here, the offset term $o_{i}$ is meant to normalize the varying sampling sizes during the data collection. In this model, we have imposed a non-structured Gaussian heterogeneity on the log intensity and refer to this model as the Poisson log-Gaussian spatial model. The Poisson log-Gaussian and log-linear models are inferentially the same. However, the MCMC implementation of the log-Gaussian model results in better convergence than the log-linear parameterization (19–21). The variance parameter $\sigma_{\eta }^{2}$ captures the non-structured spatial heterogeneity, also referred to as global spatial smoothing. The local counterpart $U_{i}$, which borrows information from neighboring observations, is modeled via conditional autoregressive (CAR) smoothing (22). That is, $U_{i}\sim \mathrm{CAR}(\sigma_{U}^{2})$, where its variance parameter $\sigma_{U}^{2}$ controls the amount of smoothing. The partial covariance of the CAR smoothing model is derived from the spatial adjacency of the contiguous spatial entities of the observations. For this, the convention is that the spatial entities that share common boundaries receive an entry of −1; those that do not share common boundaries receive 0. The unequal number of neighbors is assigned as the diagonal elements in the partial precision matrix to ensure unbiased smoothing. The coefficients $\beta_{0}$ and $\beta_{j}$ are the fixed effects, where $\beta_{0}$ is the overall intercept and $\beta_{j}$, j=1,…,J, are the mean fixed effects of the Jcovariates $x_{\mathrm{ij}}$. The random parameters $\gamma_{\mathrm{jk}}$ are the coefficients of the non-linear penalized basis functions ${\hat{z}}_{\mathrm{ijk}}=z_{\mathrm{ijk}}\Omega_{\mathrm{jk}}^{-1/2}$. We chose low-rank think-plate cubic spline basis functions $z_{\mathrm{jk}}=\{{|x_{\mathrm{ij}}-\kappa_{j1}|}^{3},\ldots ,{|x_{\mathrm{ij}}-\kappa_{\mathrm{jK}}|}^{3}\}$ based on the equally spaced fixed K knots $\kappa_{j1}<\kappa_{j2}<\ldots <\kappa_{\mathrm{jK}}$. The penalty matrix $\Omega_{\mathrm{jk}}^{-1/2}={|\kappa_{\mathrm{jl}}-\kappa_{\mathrm{jk}}|}^{3}$ penalizes the coefficients of successive basis functions to avoid over-fitting. We chose the knots to correspond with the sample quantiles of the exposure factors, considering 20 knots for each exposure factor to ensure flexibility (45).

To complete the model using Bayesian inference, we assign prior parameters to the fixed effects and hyper-priors to the variance parameters. We assigned highly diffuse priors, $\beta_{0}\sim N(0,0.001)$ and $\beta_{j}\sim N(0,0.001)$, to the fixed effects. To the random effects of the non-linear basis functions, i.e., $\gamma_{jk}∼N\left(0,\sigma_{\gamma_{jk}}^{2}\right)$, we assigned independent normal distributions. We assigned Gamma priors on the inverse of their variances, i.e., $\sigma_{\left(\cdot \right)}^{2}=\tau^{-1}$, τ~Gamma(0.5,0.00001). This is equivalent to Jeffrey’s non-informative priors (23, 24). We generated 100,000 posterior samples each for two Markov chain Monte Carlo (MCMC) simulation chains. With different starting values for each chain, the first 50,000 posterior samples were discarded. We retained every 10th simulation of the remaining 50,000 MCMC simulations for posterior summaries and were left with 10,000 simulations from the two chains to make inferences. We checked for convergence based on visual observations of the simulation trace plots. For certainty, we computed the Gelman-Rubin statistic, a formal assessment of MCMC convergence checks (25).

We implemented the model using the JAGS software via the R2JAGS package (26) of the R statistical software (27). For epidemiological analysis, we implemented two models whose differences are based on the structures of the linear predictors.


$$\text{Model}\;1:{\bar{\eta }}_{i}=\beta_{0}+U_{i}$$

$$\text{Model}\;2:{\bar{\eta }}_{i}=\beta_{0}+\sum_{j}^{J}\beta_{j}x_{\mathrm{ij}}+\sum_{j=1}^{J}\sum_{k}^{K}\gamma_{\mathrm{jk}}{\hat{z}}_{\mathrm{ijk}}+U_{i}$$

Model 1 includes only the random effects to evaluate the spatial distribution of the risk. The global smoothing or the unstructured spatial random effects term $V_{i}$ can either be simulated at each step of the MCMC, i.e., $V_{i}\sim N(0,\sigma_{\eta }^{2})$, or can be deduced as $V_{i}=\eta_{i}-{\bar{\eta }}_{i}$. Model 2 extends Model 1 by including the non-linear impacts of the exposure factors. That is, $\beta_{j}$ are the fixed effects of the exposure factors $x_{\mathrm{ij}}$, while $\gamma_{\mathrm{jk}}$ are the coefficients of the basis functions ${\hat{z}}_{\mathrm{ijk}}$. We used the proportion of nine land cover classes as our exposure factors.

An R markup file that was used to fit the Poisson log-Gaussian spatial model is provided in Supplementary File 2.

## 3 Results

**Figure 1:**
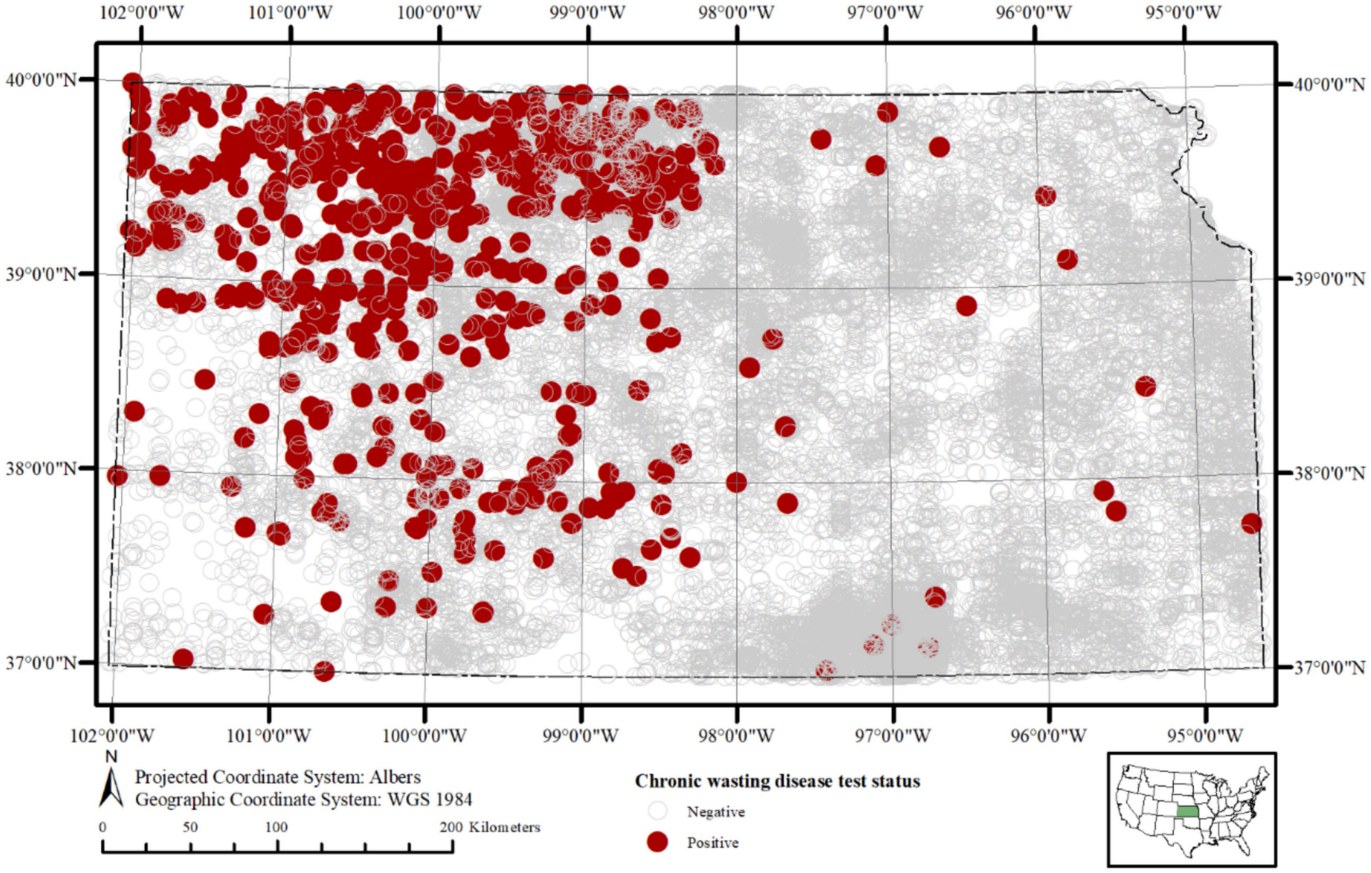
Spatial distribution of cervid tissue sampling sites for chronic wasting disease (CWD) in Kansas, United States, from 2005 to 2023. Sample locations are approximated within 5 km of deer harvest sites. Samples detecting CWD prions (*n* = 711; 3.28%) are shown as red circles, and samples where CWD prions were not detected (*n* = 20,905; 96.71%) are shown as grey open circles. This map highlights the spatial coverage of surveillance and the geographic distribution of positive and negative samples across the state and study period.

**Figure 2:**
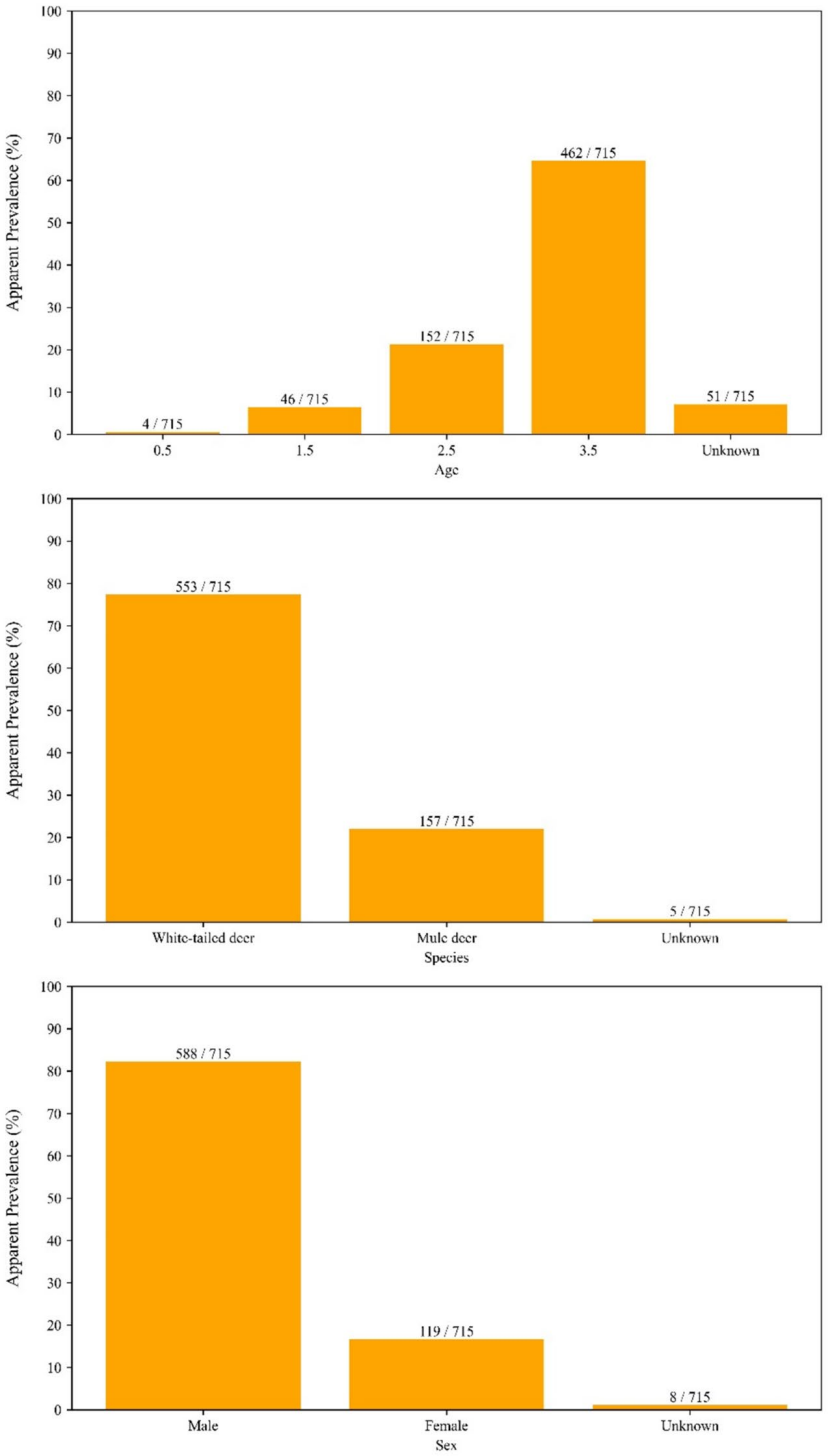
Apparent prevalence of chronic wasting disease (CWD) in cervids sampled in Kansas, United States, from 2005 to 2023, stratified by species, age, and sex. Bars show the number of samples tested in each category, and fractions above each bar indicate the proportion of samples in which CWD prions were detected. This figure illustrates how prevalence varies across demographic groups and species over the study period.

The long-term aggregated spatial pattern of CWD detected and not-detected sample locations in Kansas is present in Figure 1. The vast majority of the samples for this study (74% over the entire study period, and up to 85% in some years) came from hunter harvested (taxidermy) source, and this trend was more or less consistent over the time. A relatively higher proportion of CWD detected as well as not-detected sample locations is found distributed in a northwest to southeast gradient, with much of the detected samples concentrated in the western half of the state. Samples of CWD detected samples recorded in the central and eastern parts of the state are diagnosed among deer much more recently and were found since the year 2019. The apparent prevalence of CWD break down by different cervid species, age group, and sex are in Figure 2.

The Gelman-Rubin statistic for model convergence was noted as < 1.05 for all model parameters in both Model 1 and Model 2, indicating satisfactory MCMC convergence. The Deviance Information Criterion (DIC) for Model 1 was 1283.75, which was reduced to 1022.71 after including the habitat-level covariates in Model 2. This decrease in DIC value indicated a clear improvement in the model fit and implied the importance of the habitat factors as potential predictors of CWD risk over the geographic extent. When exponentiated, the intercept (exp(*β*_0_) = 0.747) reflects the overall risk of CWD infection, which far exceeds the crude estimate of 0.032. It further suggests that our model, which accounts for fixed and non-linear random effects as well as spatial random effects, effectively captures the variation in CWD risk across the study area.

**Figure 3:**
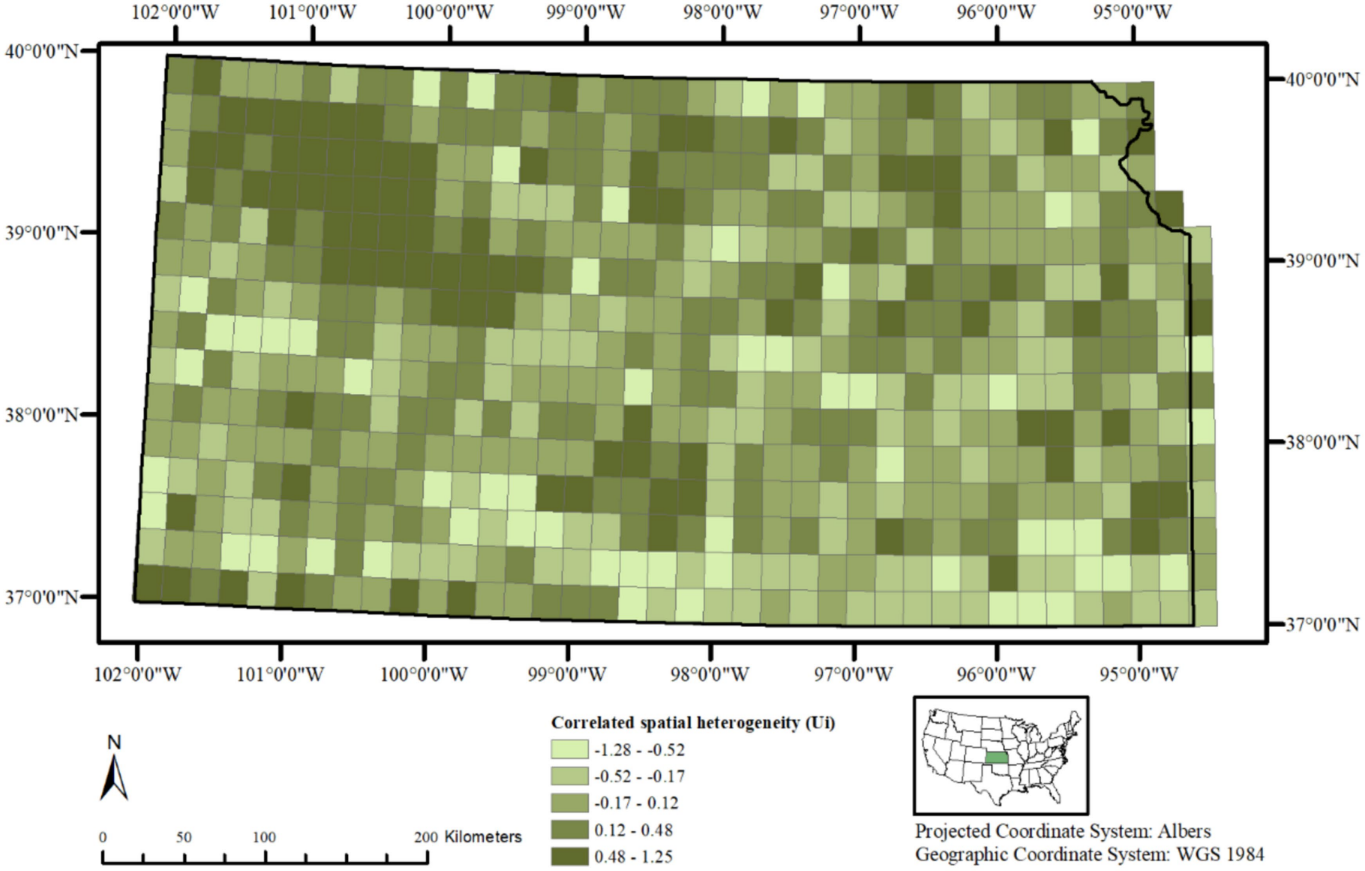
Posterior mean of correlated spatial heterogeneity (Uᵢ) in chronic wasting disease (CWD) risk across 20 km^2^ grid cells in Kansas, United States, based on surveillance data from 2005 to 2023. Higher values in the northwest indicate local clusters of elevated risk among neighboring areas.

**Figure 4:**
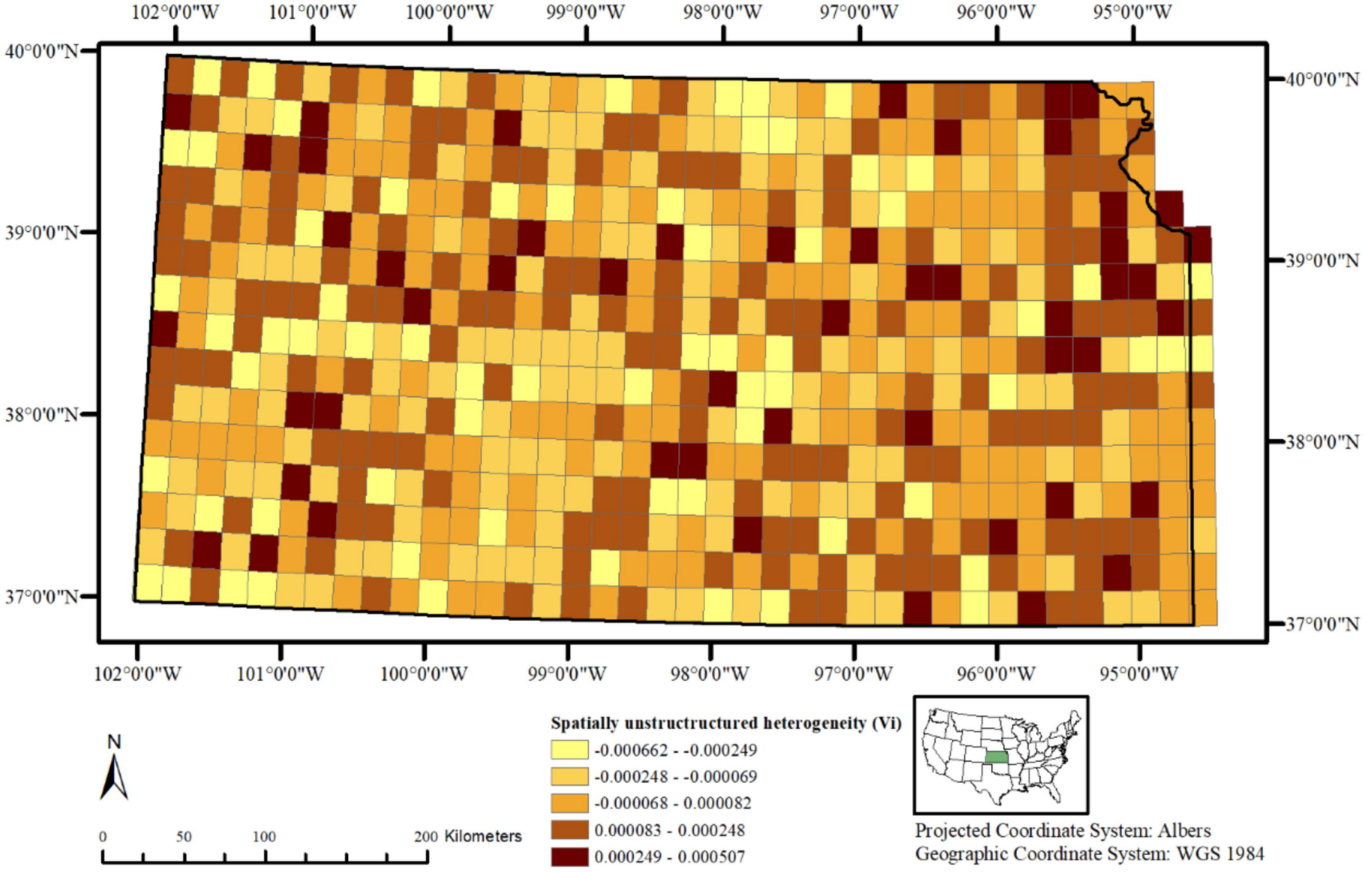
Posterior mean of unstructured spatial heterogeneity (Vᵢ) in CWD risk across 20 km^2^ grid cells in Kansas, United States, from 2005 to 2023. Values vary locally without broad clustering, indicating independent deviations from spatially structured risk.

A choropleth map of the posterior means of spatially structured heterogeneity $U_{i}$ (Figure 3) revealed two independent but contiguous areas in the northwestern and southcentral portions of the state with closer values, indicating same or similar underlying spatial processes driving CWD distribution in these clusters. A choropleth map of the posterior mean of non-structured spatial heterogeneity $V_{i}$ shows a heterogeneous distribution of lower and higher values throughout the state (Figure 4) without any clusters, indicating that in some of the 20 km^2^ grid cells, there are habitat-level factors that influence CWD presence more than others while global trends are absent.

**Figure 5:**
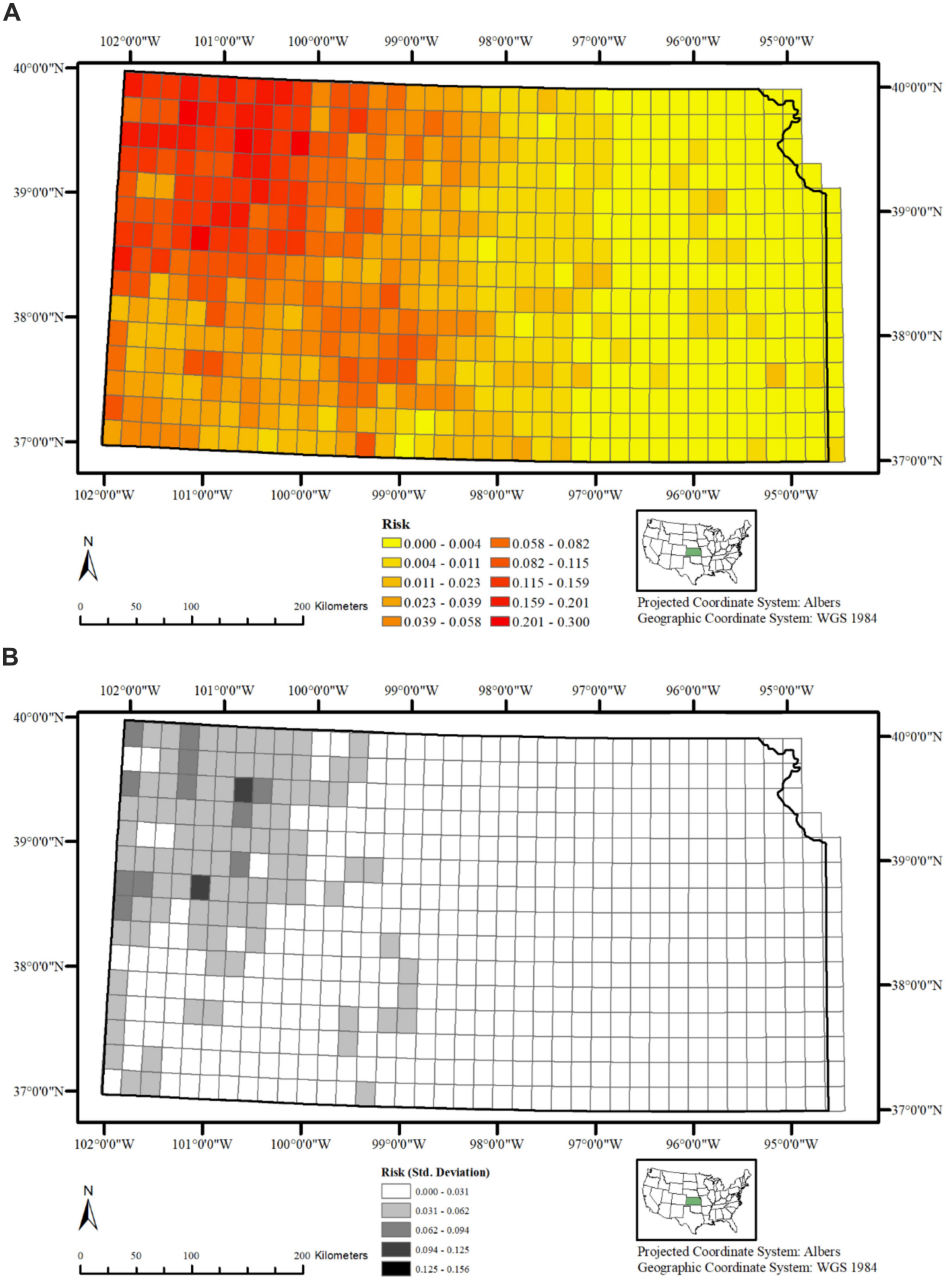
**(A)** Posterior mean of CWD risk across Kansas, United States, from 2005 to 2023, showing highest predicted risk in the northwest, decreasing toward central and eastern regions. **(B)** Standard deviation of the posterior mean CWD risk across Kansas, United States, from 2005 to 2023, indicating uncertainty in grid-cell level predictions; most areas show low variability.

The variation in CWD risk within a given 20 km^2^ grid cell is plotted in Figure 5A. The northwestern areas of the state, followed by the southern regions, clearly face a higher risk compared to the eastern part, while the central region experiences a moderate level of risk. A general sense of directionality of disease spread is visible in this plot, indicating that the infection among cervids decreases in intensity when moving away from the northwestern areas toward the central and eastern portions. The uncertainty (Std. dev) associated with the posterior mean of risk estimates are presented in Figure 5B, and indicate no major concern except for the observance of higher deviations in two 20 km^2^ grid cells in the northwestern portion of the state. This suggests that the model predicts the risk of CWD presence in the study region to a satisfactory extent.

**Figure 6:**
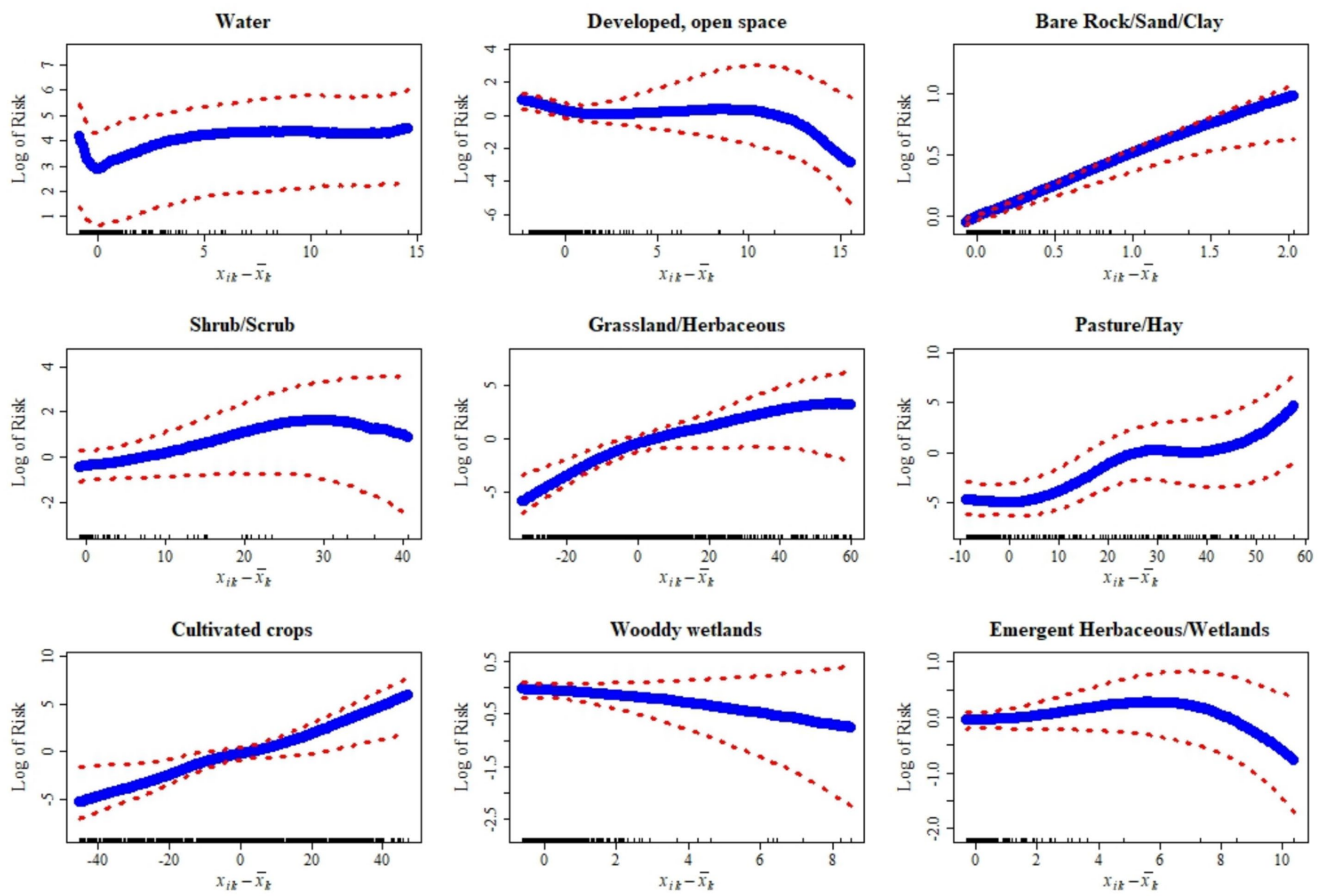
Relationships between deviations $\left(x_{\mathrm{ik}}-{\bar{x}}_{k}\right)$ of habitat-level covariates from their mean and the log risk of chronic wasting disease (CWD) along with their corresponding 95% credible intervals (red dotted lines) of CWD, in 20 km^2^ grid cells across Kansas, United States, based on surveillance data from 2005 to 2023. These plots show how variation in land cover and habitat features is associated with spatial risk.

The following results describe statistical associations between land cover covariates and the predicted probability of CWD presence. These associations should not be interpreted as causal effects, since the model does not establish mechanistic pathways linking habitat features to disease dynamics. Instead, the observed patterns may reflect underlying ecological, host, or surveillance processes not directly measured in this study. The relationships between habitat covariates and the risk of CWD presence display a range of patterns, from strong and consistent to subtle and uncertain (Figure 6). Some land cover types, like Bare rock/Sand/Clay and cultivated crops, show a clear and steep increase in risk as their presence grows, indicating a strong and well-defined association with higher disease occurrence. Pasture and hay follow a similar trajectory, with risk rising sharply in a non-linear fashion. However, this relationship carries greater uncertainty at more extreme values, suggesting variability in their effect. Other covariates exhibit more moderate or complex patterns. Shrub and scrub areas, for example, are associated with a gradual increase in risk, though the strength of this relationship becomes less certain as their extent increases. Grassland and herbaceous cover show a modest rise in risk that tends to flatten at higher levels, reflecting a diminishing return effect. Woody wetlands reveal a nuanced pattern: they appear to reduce risk at lower values but contribute to increased risk as they become more prominent, with the overall relationship marked by considerable uncertainty at the extremes.

In contrast, the presence of water bodies like lakes and streams seems to have only a marginal impact. The risk rises slightly at first but quickly plateaus, indicating a limited and stable influence. Developed open space shows a subtle negative relationship, where risk decreases slightly and then levels off, suggesting that such areas may slightly deter disease presence. Emergent herbaceous wetlands have the least impact, with a nearly flat relationship that hints at minimal influence on risk, regardless of their abundance. Collectively, these patterns reflect the diverse ways in which different land cover types shape the spatial distribution of CWD, with some exerting strong, direct effects and others contributing little or introducing greater uncertainty.

## 4 Discussion

This study has analyzed the spatial variation of CWD risk in Kansas using an innovative modeling approach that accounts for rare occurrence of positive events, and imbalance in class outcomes that are typically seen with wildlife disease surveillance datasets. Our Poisson log-Gaussian spatial model explicitly accounted for these shortcomings by incorporating an offset term to normalize varying sample sizes, modeling both structured and unstructured spatial heterogeneity to borrow strength across neighboring areas, and including non-linear covariate effects to capture complex habitat–disease relationships. This framework allowed us to disentangle true spatial risk patterns from sampling artifacts, improve model convergence and fit, and generate robust risk estimates despite the inherent imbalances of opportunistic data (19, 22, 28). By doing so, our framework disentangled true spatial risk patterns from sampling artifacts, improved model convergence and fit, and generated robust risk estimates despite data limitations.

We excluded temporal random effects in our modeling framework because spatial units in Kansas were sampled inconsistently across years, making temporal structures weakly identifiable and overly influenced by priors rather than data (15, 16). Given that our covariates were relatively stable over time and the study’s objective was to characterize long-term spatial patterns, temporal terms would have added unnecessary complexity without improving inference (17). Their exclusion also reduced computational burden and convergence issues common in hierarchical models with sparse, zero-inflated data (18). Accordingly, our results should be interpreted as reflecting long-term aggregated spatial patterns of disease risk, not short-term fluctuations. This time-aggregated perspective emphasizes persistent spatial heterogeneity and enduring geographic hotspots that can inform long-term management, but it does not capture year-to-year changes, seasonal dynamics, or the timing of disease spread, which would require more temporally balanced data.

Together with our prior modeling of this data (29), the random spatial factors, and the habitat-level covariates included in our present analysis help explain variability in the surveillance data, indicating that understanding how these covariates interact with disease risk can provide some insights for development of CWD management options (e.g., disease hot spots, impact of hunter-deer interaction, movement pathways, etc.) (30–32). Further, this study has calculated various spatial metrics, which, when plotted, allow us to visualize how the risk of CWD presence fluctuates across Kansas. It also demonstrates how different covariates influence this spatial distribution through measures of both correlated and uncorrelated spatial heterogeneities, while identifying specific non-linear patterns in habitat-level covariate associations with CWD risk.

Correlated spatial heterogeneity $U_{i}$ (Figure 3) implies that the variations in CWD presence within the different 20 km^2^ grid cells are not independent but are spatially correlated. In other words, the outcome in neighboring areal units is likely to be similar due to one or more underlying spatial process or structure. The correlated heterogeneity $U_{i}$ in this study, based on Model 2, shows a noticeable cluster on the northwestern portion of Kansas, extending toward the southcentral region. A second cluster of higher values of $U_{i}$ is also prominent in the southcentral region, although the size of this cluster is relatively small. These results are consistent with prior work showing that CWD often clusters within localized foci and spreads outwardly, influenced by deer movement and landscape connectivity (30,32). These clusters indicate that the spatial processes influencing CWD presence among cervids such as deer dispersal, social group interactions, or environmental reservoirs of prions in these areas is continuous, and spreads over space, affecting the nearby locations similarly (33, 34). Further, the values surrounding these two clusters do change abruptly, and high and low-value grid cells are randomly scattered throughout the rest of the study area, often close to each other. This suggests that, while our model successfully identifies regions with similar habitat characteristics that influence CWD presence, there are still unknown or yet to be analyzed factors, which may include soil mineral composition (34, 35), land use intensity (46), or human mediated transport (47) that contribute to the spatial distribution of CWD.

The uncorrelated heterogeneity or spatially unstructured heterogeneity $V_{i}$ (Figure 4) helps identifying variations in the risk of CWD presence at different 20 km^2^ grid cells independent of each other, without any spatial correlation with neighboring spatial units. In other words, those grid cell areas with higher values indicate that their CWD presence is influenced by habitat-level factors that are unique to them alone, rather than those that are continuous over space. While $V_{i}$ values widely vary across the study area, clear clusters are absent. This suggests that despite the presence of global trends, there are also local deviations from these global trends, where potentially some habitat-level factor(s) influence the variation in each grid cell differently. Similar findings have been reported in Wisconsin and Illinois, where fine-scale land use and habitat features influenced CWD distribution in ways not fully explained by global spatial trends (36, 37). A further identification of such spatially varying influence of habitat factors would require additional analysis with a different model specification and incorporation of soil chemistry, hydrology, and deer density estimates, which have been implicated in prion persistence and disease spread elsewhere (38, 39).

A plot of exponentiated intercept additively calculated along with $U_{i}$ and $V_{i}$ reflects the overall risk of CWD presence. When plotted for each grid cell, it is evident that the risk of CWD presence varies greatly over Kansas (Figures 5A,B), with higher risk of CWD presence in the northwestern portion of the state and progressively lower risk in the remainder areas; Rivers and streams in Kansas generally flow from northwest to southeast, suggesting that the disease is spreading along these waterways, which deer use for movement and foraging. Such influence of rivers and stream corridors in the spread of CWD have previously suggested by others (34, 40). Although the overall statistical effect of water bodies on CWD risk is marginal and levels off quickly, the directional flow and ecological role of rivers may still promote localized transmission by connecting habitat patches and concentrating deer activity, highlighting the complex multi-factorial nature of landscape-disease interactions.

While eastern Kansas could be expected to show higher risk if it were simply the advancing edge of the invasion front from the northwest, our spatial model results indicate otherwise. The correlated heterogeneity ($U_{i}$) identified persistent clusters of higher risk in the northwest and central regions but similar clusters were not found in the east. Likewise, the uncorrelated heterogeneity ($V_{i}$) showed no localized clusters in the east that would suggest recent establishment of the disease. Instead, the posterior risk surface revealed consistently lower risk estimates in the eastern portion of the state. Such patterns suggest that the current distribution of CWD risk in the state is not explained by directional spread alone but is relatively strongly influenced by habitat-level features, such as land cover, fragmentation, or hydrography, which may limit disease persistence and spread in the east.

The relatively higher standard deviation observed for the posterior mean of risk in the northwestern portion of the state compared to the other areas is unexpected, given that there is adequate representation of surveillance data in this area. While the deviations are not particularly concerning since they primarily fall within the lower ranges, they may indicate potential deficiencies in our model specification. This also suggests that we might need to incorporate additional habitat or environmental covariates (such as soil type or elevation) to better account for the variability in the current dataset (39, 41, 46). Further, our surveillance dataset is unbalanced with overrepresentation of zeros (i.e., zero inflation), which may have contributed to the higher levels of deviation in the areas where they were observed.

Habitat-level covariates, like those analyzed in this study and available in the National Land Cover Dataset, serve as valuable proxies for understanding the ecology of wildlife diseases. In this study, we focused on habitat-level vegetation and land cover covariates, which have independently yielded reliable model predictions of risk and spatial heterogeneities. Factors like habitat fragmentation (e.g., connectivity, edge density), soil properties (29), and hydrographic features in the landscape however may further influence CWD dynamics by impacting deer density and environmental conditions that support prion survival. Including such covariates from diverse habitat themes will potentially yield even better predictions of CWD risk over the Kansas region and may allow for more direct interpretations of their risk to CWD. One approach to this could be the use of spatially varying coefficient models, that accounts for relative differences in covariate importance over spatial and/or spatiotemporal extents (42, 43). Further, the patterns of habitat-level covariate association with CWD described herein (Figure 6) indicate that some, if not all of them could be evaluated as potential drivers or risk factors for the disease. Two modeling considerations are important for such analysis. First, using a Bernoulli or binomial approximation to model individual-level CWD data may provide more accurate associations. Second, if there is class imbalance in the surveillance dataset outcomes, it will be necessary to apply a data balancing step prior to modeling.

Land cover/land use features quantified in NLCD is fairly consistent over time and are available at a relatively fine spatial resolutions which make them particularly useful for designing and implementing potential disease management strategies. For instance, areas identified as high-risk in this study could be prioritized for stricter regulation of hunting practices and carcass transport/disposal, given that movement of infected carcasses is a known pathway for disease spread (33). While targeted deer population reduction through sharp-shooting is not currently an adopted strategy in Kansas, such approaches have been applied in other jurisdictions to limit CWD spread and prevalence (36, 44). If such measures were ever considered, the results of this study could guide managers in selecting spatially targeted locations where interventions would likely be most effective. In this way, the integration of consistent, fine-resolution land cover data with spatial risk modeling provides a science-based framework to support both current and potential future CWD management strategies.

## 5 Conclusion

This study highlights the variations in chronic wasting disease (CWD) risk across Kansas and identifies key habitat-level factors influencing disease risk in the state. While previous work and surveillance data indicate a temporal spread of CWD from Colorado into Kansas, our findings suggest that the present-day risk distribution is not solely explained by directional spread alone. Instead, CWD risk is now strongly associated with specific habitat characteristics and exhibits persistent clusters of higher risk in the northwestern, western, and central regions, with relatively lower risk in the east. Estimating the spatial distribution of wildlife diseases using surveillance data presents challenges, often requiring data-driven models that account for various complexities. The Poisson log-Gaussian model developed in this study performed effectively and was specifically designed to address rare events, spatial and temporal sampling inconsistencies, and data imbalances. This model has the potential to be adapted for analyzing similar disease datasets that are based on surveillance efforts with non-uniform effort in sampling.

## Data Availability

The raw data supporting the conclusions of this article will be made available by the authors, without undue reservation.
