# Supplementary material for: Evaluation of spatial variation in chronic wasting disease risk with Bayesian Poisson log-Gaussian model
Source: Front Vet Sci. 2025 Nov 7;12:1568468. doi: 10.3389/fvets.2025.1568468 (PMC12636040; doi:10.3389/fvets.2025.1568468)
Supplement: Supplementary file 1 [file Supplementary_file_1.docx]

**Supplementary File 1**

**Exploratory Bayesian models**

| Model | Parameters | DIC | Gelman-Rubin statistic |
| --- | --- | --- | --- |
| 1 | Disease status ~ Bernoulli (Sex + Space) | 1621 | 1.06 |
| 2 | Disease status ~ Bernoulli (Age + Space) | 1623 | 1.06 |
| 3 | Disease status ~ Bernoulli (Age + Sex + Space) | 1621 | 1.05 |
| 4 | Disease status ~ Bernoulli (Age + Sex + Source + Space) | 1627 | 1.05 |
| 5 | Disease status ~ Bernoulli (Space + Land cover) | 1516 | 1.06 |
| 6 | Disease status ~ Bernoulli (Age + Sex + Space + Land cover) | 1514 | 1.08 |
| 7 | Disease status ~ Bernoulli (Age + Sex + Source + Space + Land cover) | 1517 | 1.10 |

The disease status $Y_{i}$ was modeled assuming a Bernoulli approximation, $Y_{i}\sim Bernoulli (p_{i})$ with a logit link function $logit\left( p_{i} \right)= \beta_{0}+ \beta_{(1,..n)}x_{i(1,..,n)}+ u_{i}+v_{i}$ , such that $p_{i}=\frac{exp (\eta_{i})}{1+exp (\eta_{i})}$, where $\eta_{i}=\beta_{0}+\beta_{(1,..,n)}x_{i(1,..,n)}+u_{i}+v_{i}$. The spatial effects (space) in the data were modeled using two components, $u_{i}\sim CAR \left( T_{u} \right)$, and $v_{i}\sim N(0, T_{v}^{-1})$ for the spatially structured random effect, and unstructured random effect, respectively. Land cover (%) estimates were calculated for each observation by summarizing values within 5km circular buffer zone. Non-informative priors were utilized for the fixed effects, and CAR (conditional autoregressive) and Gaussian for the two spatial effects, respectively. Two chains of all models were run for 50,000 iterations each with 10,000 burn-in runs. Sample origin (taxidermy, hunter-harvested, biologists, etc.), and demographic factors (age, sex) did not affect the model performance by >3 units, and these models did not converge satisfactorily. Additionally, upon visual inspection the spatial maps of posterior means for the risk parameter did not vary to a marked extent, and therefore they were not considered as independent covariates.
